# Self-Selection Bias: An Essential Design Consideration for Nutrition Trials in Healthy Populations

**DOI:** 10.3389/fnut.2020.587983

**Published:** 2020-11-10

**Authors:** Lauren M. Young, Sarah Gauci, Andrew Scholey, David J. White, Andrew Pipingas

**Affiliations:** Centre for Human Psychopharmacology, School of Health Sciences, Swinburne University, Hawthorn, VIC, Australia

**Keywords:** self-selection bias, study design, nutrient status, randomized controlled trials, nutrient effects

## Abstract

Many researchers have identified the issue of self-selection bias hindering the ability to detect nutrient effects in healthy populations. However, it appears that no effort has been made to mitigate this potential design flaw. By recruiting individuals on the basis of pre-trial dietary intake, the Memory and Attention Supplementation Trial aimed to capture a cohort of participants with a wide variety of dietary intake, thus increasing the likelihood of a diverse range of nutrient status. This perspective specifically examines the profile of these trial volunteers and in doing so, we present the first empirical evidence of self-selection bias when recruiting healthy volunteers for a randomized controlled trial of a nutrient-based supplement. These findings support the anecdotal proposal that traditional recruitment methods inherently attract trial volunteers who are vastly unrepresentative of the population and threatens the generalizability of this field of research. Alternative approaches to recruitment, including *a-priori* screening for baseline diet quality and nutrient status, are discussed as essential design recommendations to ensure accurate interpretation of nutrient effects within the context of baseline participant characteristics.

## Introduction

Nutrition science has revealed numerous relationships between diet quality and systemic health. Research over the past two decades has seen an increased focus on the role of diet quality and nutrient intake for psychological well-being (1). Cross-sectional and epidemiological studies have established that higher circulating levels of essential nutrients, including vitamins B, C, D, and E, is associated with reduced risk of age-associated cognitive impairment (2–5) and an increased ability to cope with psychological demand and stress (6). Within the context of an aging population, the shift in dietary profile of Western countries in the twenty-first century, characterized by an increase in processed foods, has led to an increased concern for the long term impact of poor nutrition on the brain (7–9). However, the legitimacy of this concern remains uncertain with the majority of evidence garnered from studies utilizing memory-based methods of dietary assessment (10).

Despite these unknowns, there has been an increased use of dietary supplements including nutraceutical formulae. This is due to the commonly held belief that supplements may compensate for nutrient insufficiencies not met through diet alone (11, 12). While there is encouraging research for a number of specific nutrients (B Vitamins, Vitamin C, Vitamin D, essential fatty acids) for aspects of mental health, the majority of dietary supplementation research has been in clinical populations or those with a frank nutrient deficiency (12–14). Whether such nutrient-based supplements impart any benefit to cognition or mental health in healthy, non-clinical populations is currently unclear. One purported explanation for the inconsistencies in findings across studies of healthy participants is that the methodology applied in such trials is flawed, and may undermine the effect of nutrient supplementation. Herein we focus on one aspect of trial methodology, the “healthy participant” effect (15–17), providing evidence of self-selection bias derived from insights learned from our supplementation trial in healthy participants.

## The Design Flaw

**Figure 1:**
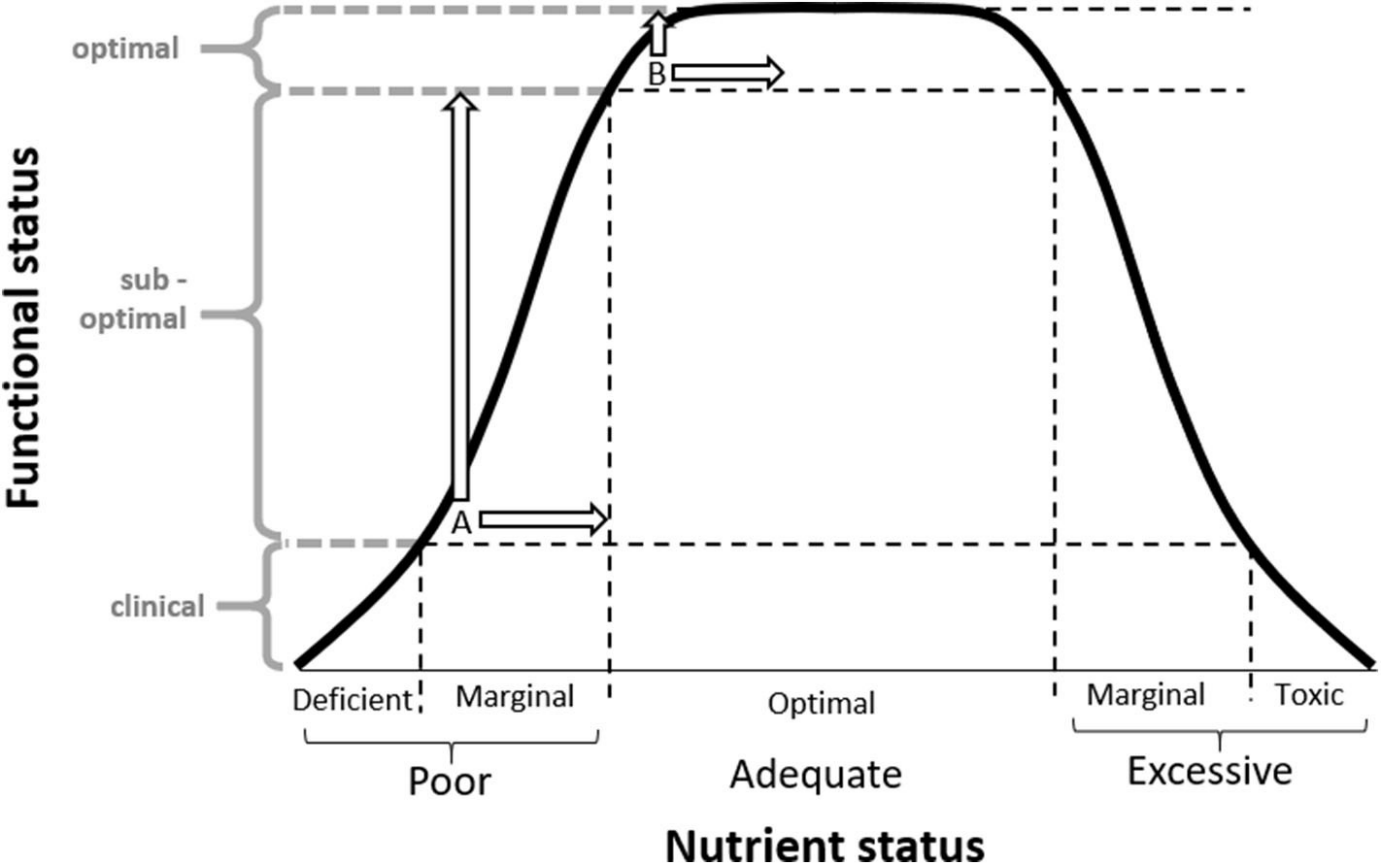
Inverted “U” hypothesis diagram adapted from Scholey (14) depicting the relationship between nutrient status and functional status, and how an individual's response to supplementation is dependent on their baseline status. When an identical dose is provided, individuals with marginal insufficiencies at baseline (A) will produce a greater functional response in comparison to those with “optimal” nutrient intake at baseline (B). Supplementation trials (including the MAST study) often attract participants with “optimal” nutrient intake at baseline (B), which may limit the ability to measure a potential benefit on functional status. © Scholey 2017, reprinted with permission.

One potential design limitation in supplementation trials was raised by Morris and Tangney in 2011 (18). They proposed that the participants who volunteer for supplementation trials may already have “optimal” nutrient status. If we assume that nutrient status can be represented by an inverted “U” relationship, an individual's behavioral response to supplementation will be dependent on their baseline status (Figure 1) (19). Therefore, individuals with “optimal” nutrient status are unlikely to gain further health benefits from nutrient supplementation (Intake B) (18). In contrast, a cohort with “sub-optimal” status (or “nutrient insufficiency”) have a greater potential for detectable functional improvement through supplementation (Intake A). Elevated baseline nutrient status in trial participants has the potential to explain the plethora of research reporting null effects of nutrient supplements in a clinical trial setting, despite the evidence that increased nutrient status is related to better health outcomes. Prevention trials, however, rarely consider participants' baseline nutrient status in trial design (19, 20). Anecdotally, many researchers report that volunteers of prevention trials are typically health literate, and thus may be less likely to have “sub-optimal” nutrient intake prior to supplementation (11, 18, 21). This has been highlighted by the limited variability in dietary patterns and narrow range in nutrient status which often precludes *post-hoc* subgroup analysis.

If supplementation trials in non-clinical groups attract health conscious individuals, this has important ramifications for the field of nutrition science. Firstly, it suggests that such cohorts are not representative of the population, limiting the generalizability of (positive or negative) results. Secondly, if participants already have elevated nutrient status at baseline, any potential effects of nutrient-based supplements will be more difficult to detect as there is limited scope for improvement. Taken together this fairly simple methodological issue could undermine a whole field of research. Yet, to date, no research has attempted to address this issue.

## Addressing Self-Selection bias in the Memory and Attention Supplementation Trial

The opportunity to test this hypothesis of self-selection bias in trial participants was considered in the design of the Memory and Attention Supplementation Trial (MAST) conducted in Melbourne, Australia from June 2018 to January 2020 (NCT03482063). The primary aim of the trial was to investigate the efficacy of a multinutrient supplement on neurocognitive function and mood in healthy, middle-aged adults. The study utilized a novel recruitment strategy, whereby volunteers were screened via interview to assess their diet quality prior to enrolment in the study. The study aimed to recruit a cohort comprising 50% participants with an “optimal” diet and 50% with a “sub-optimal” diet. The dietary screen provided an umbrella view of overall diet quality and has previously been validated as a predictor of nutrient status (22, 23). This dietary screen has been described elsewhere (24). Briefly, in order to be classified as having an “optimal” diet, volunteers needed to consume—a high intake of fruit, vegetables, legumes, olive oil, nuts, and a lower intake of processed foods (sweets, chocolate, cakes, processed meats, sugar-sweetened beverages). We have recently reported that the dietary screening tool accurately distinguished key nutrient levels as determined by blood biomarkers (24). Importantly, randomization was stratified by diet quality such that each diet subgroup had equal chance of being allocated to placebo or active treatment. To our knowledge, this is the first randomized controlled trial of a nutrient-based supplement which recruited healthy participants on the basis of their initial dietary status. Using diet quality as a proxy for nutrient status ensured a cohort with a broad range of dietary intake, thus increasing the likelihood of a diverse range of nutrient status.

While the analysis of the intervention trial itself is ongoing, the baseline characteristics of the study provide the first empirical evidence of self-selection bias in nutrient-based supplementation studies. In order to randomize 140 participants to study treatment (50% with a “sub-optimal” diet), 501 volunteers completed the preliminary screen via telephone which included the aforementioned diet quality assessment. The process of recruitment revealed that of the 461 volunteers with dietary screen information, 74.4% met the criterion for an “optimal” diet indicative of elevated nutrient status. This is particularly noteworthy when contrasted with the most recent population statistics reporting that only 5.4% of Australian adults meet the current recommendations for both fruit and vegetable intake (25). In other words, despite more than nine out of 10 Australians adhering to a diet that would be considered “sub-optimal,” the ratio was <3 out of 10 for volunteers for this clinical trial. We have no reason to believe that this study is unusual regarding initial recruitment, however it overwhelmingly attracted the unrepresentative group of individuals with an “optimal” diet. In total, 227 volunteers were excluded from the study as their diet quality was considered “optimal” and the cohort of participants with an “optimal” diet had already been filled. Having this quota set at 50% for our “sub-optimal” diet group allowed the study to recruit individuals who would not typically be captured in clinical trials and due to adherence to a poorer quality diet were more likely to have “sub-optimal” nutrient status.

Upon enrolment in the study, participants' biochemical markers were assessed to determine whether meaningful differences in terms of circulating levels of nutrients could be ascertained from this relatively simple measure of diet quality. This data has been presented elsewhere (24). We found that individuals with an “optimal” diet were older, had a lower body mass index, significantly higher circulating levels of vitamin B6, red cell folate, and lower levels of saturated fatty acids than individuals with a “sub-optimal” diet. Further, individuals with an “optimal” diet had significantly higher intake of vitamin E, magnesium, zinc, and fiber, as compared to 24 h diet recalls. Collectively, these findings support the notion that those classified as having an “optimal” diet were characteristically different to those classified as having a “sub-optimal'' diet.

If this study is representative of clinical trials investigating healthy participants, the abundance of volunteers classified as having an “optimal” diet confirms the anecdotal proposal that individuals who volunteer for nutrient-based supplementation trials may be characteristically different from “non-participants.” This would inherently limit the ability to generalize the findings of these studies to the general population.

## Discussion

Experimental bias when recruiting from healthy populations could be attributed to a number of factors. Firstly, volunteering for a randomized controlled trial often involves a significant time commitment from participants, with relatively little monetary compensation and no guarantee of gain (due to chance of allocation to placebo). This self-selection often attracts individuals who already have an interest in research or the subject area and therefore are looking to improve their health. Further, it is commonly noted that, compared with the general population, clinical trial volunteers are often well-educated with higher socioeconomic status, allowing them to sometimes forgo income for the time to participate in their common interest in research. Additionally, individuals with poor diet quality may be willing to volunteer, but their diet has already impacted their health such that they no longer meet eligibility criteria for clinical trials. For example, poor diet quality is associated with increased likelihood of requiring treatment for a mood disorder, high intake of alcohol, diabetes, uncontrolled hypertension and existing consumption of vitamin supplements (8, 9, 26); all of which are common exclusion criteria for trials studying healthy participants.

Almost a decade on from the Morris and Tangney's proposal of “optimal” nutrient status hindering supplementation trial research (18), the evidence presented here provides the first empirical evidence that this design flaw of self-selection bias does in fact exist. Consequently, the persistent use of traditional recruitment techniques in nutrient supplementation research has likely resulted in the continued collection of data from individuals who are characteristically different from the general population. As we speculate that the health effects of supplementation are only marginal, adherence to a nutrient-rich diet has the potential to undermine these effects entirely, thus the resulting research is vulnerable to Type II error. In contrast, those who we suspect may benefit most from supplementation—those with poorer nutrient status—may be precluded from this type of research due to comorbidities that render them ineligible within the strict confines of inclusion criteria. This is an unfortunate by-product of randomized controlled trials prioritizing the reduction of inter-individual variability over representation of the general population.

Nevertheless, it would be remiss to not consider the potential bias in the presented example. The screening tool used to classify “optimal” and “sub-optimal” relies on the participant recalling their own perception of their usual dietary intake and is not an objective measure of dietary intake (10). Moreover, the original validation paper awarded five additional points for dietary supplement use. This may have biased the initial validation since supplement users tend to consume a healthier diet and be more physically active than non-users (27). It should be noted however that the use of supplements was an exclusion criterion in the present study so we can be reasonably confident that this did not skew the diet screening scores. While memory-based methods of dietary assessment will never replace objective measures of assessment such as biochemical indices, in the case of the present study, the diet quality screen served as a practical, cost-effective method to classify participants in an attempt to capture participants with a broad range of nutrient profiles. We were then able to show that the diet groups classified in this way differed across a number of biochemical measures of nutrition status (24), and the “optimal” group was three times more likely to volunteer to participate than the “sub-optimal'' group.

Further, one must be cautious when interpreting nutrient status from self-report measures of dietary intake (28). While the diet groups differed across a number of blood biomarkers, the tool did not distinguish across all B group vitamins (24). In addition, the synergistic (29) and “rate-limiting” effects of nutrients highlight that the inverted “U” hypothesis of nutrient status may be an oversimplified model which could greatly hinder nutrition science. The assumption that “more is better” in terms of nutrient intake is problematic when we consider the vast inter-individual differences in metabolism and physical activity levels. Failure to acknowledge these unique differences may have resulted in over attributing causal effects of individual nutrients.

Finally, this study is but one example of self-selection bias in a small sample of participants. Other factors that relate to diet quality, including socioeconomic status and education, may also confound participant selection. Cohort studies which require long term follow up may have heightened susceptibility to this bias due to more onerous requirements for participation that would only exacerbate the “healthy participant” effect. Yet, self-selection bias continues to be overlooked in nutrition trial design. The evidence presented in this perspective highlights the urgency to address this issue.

## Directions for Future Research: A-Priori Screening

In the face of this major methodological flaw, research continues to utilize convenience sampling with a complete disregard for how participant selection may undermine the research. The acceptance of this design limitation introduces uncertainty to the conclusions of previous nutrient-based supplementation trials in healthy populations. In order to overcome this and generalize results of clinical trials within the field of nutrition science, it is recommended that future studies prioritize representation of the population from which the sample is drawn. While alternative screening approaches such as the one used in the present study may be time consuming, it will help us understand the effect of nutrient-based supplements across the broader population and therefore, identify subgroups who are most likely to benefit. Future studies should consider *a priori* screening for diet quality to better characterize potential participants prior to inclusion in their trials. The use of biochemical markers of nutrient status is essential to ensuring that the efficacy of the supplement in question is interpreted within the context of baseline status (19).

## Conclusion

Randomized controlled trials of supplements in healthy populations are critical if we seek to identify interventions that could improve the mental health of those with sub-clinical symptomology and prevent against future brain disorders. This work emphasizes that unless specific recruitment is used to target a broad range of the population, traditional recruitment strategies inherently attract participants who are health conscious and may have elevated nutrient status prior to supplementation. Self-selection bias has the potential to significantly undermine any nutrient effects and render the field of nutrition science vulnerable to Type II error. While seemingly obvious, hundreds of studies of nutrient effects continue to overlook baseline diet and nutrient status (19). Previously anecdotal, this study provides the first empirical evidence of self-selection bias when studying healthy participants. Acceptance of this design flaw emphasizes the urgent need to factor baseline participant profiling into trial design and data interpretation.

## Data Availability Statement

The original contributions presented in the study are included in the article, further inquiries can be directed to the corresponding author/s.

## Ethics Statement

The studies involving human participants were reviewed and approved by Swinburne University Human Research Ethics Committee. The patients/participants provided their written informed consent to participate in this study.

## Author Contributions

LMY, AS, DJW, and AP conceived the study and had significant input into design. LMY and SG were largely responsible for recruitment and data collection. LMY drafted the initial version of the manuscript. All the authors participated in critical revision of the manuscript. All authors contributed to the article and approved the submitted version.

## Conflict of Interest

AS, DJW, and AP have received research funding, honoraria, and conference support from the nutrition and supplement industry. The remaining authors declare that the research was conducted in the absence of any commercial or financial relationships that could be construed as a potential conflict of interest.
